# Transmissibility and Persistence of the Plasmid-Borne Mobile Colistin Resistance Gene, *mcr-1*, Harbored in Poultry-Associated *E. coli*

**DOI:** 10.3390/antibiotics11060774

**Published:** 2022-06-07

**Authors:** Hassan Al Mana, Alreem A. Johar, Issmat I. Kassem, Nahla O. Eltai

**Affiliations:** 1 Biomedical Research Centre, Microbiology Department, Qatar University, Doha 2713, Qatar; h.almana@qu.edu.qa; 2 Research and Development Department, Barzan Holdings, Doha 7178, Qatar; ajohar@barzanholdings.com; 3 GA Centre for Food Safety, Department of Food Science and Technology, University of Georgia, Griffin, GA 30609, USA; issmat.kassem@uga.edu

**Keywords:** colistin, *mcr-1*, *E. coli*, fitness, transmission, biofilm

## Abstract

Colistin, a last-resort antibiotic, is used to treat infections caused by multi-drug-resistant Gram-negative bacteria. Colistin resistance can emerge by acquiring the mobile colistin gene, *mcr-1*, usually plasmid borne. Studies on *mcr-1* and its transmissibility are limited in the Middle East and North Africa (MENA) region. Here, we investigated the occurrence of *mcr-1* in 18 previously collected *Escherichia coli* isolates collected from chicken samples in Qatar; whole-genome sequencing was performed to determine the location (plasmid-borne and chromosomal) of *mcr-1* in the isolates. Additionally, we assessed the transmissibility of plasmid-borne *mcr-1* and its cost on fitness in *E. coli* biofilms. Our results showed that the *E. coli* isolates belonged to different sequence types, indicating that *mcr-1* was occurring in strains with diverse genetic backgrounds. In silico analysis and transformation assays showed that all the isolates carried *mcr-1* on plasmids that were mainly IncI2 types. All the *mcr-1* plasmids were found to be transmissible by conjugation. In biofilms, a significant reduction in the number of CFU (≈0.055 logs CFU/mL) and colistin resistance (≈2.19 log CFU/mL) was observed; however, the reduction in resistance was significantly larger, indicating that the plasmids incur a high fitness cost. To our knowledge, this is the first study that investigates *mcr-1* transmissibility and persistence in Qatar. Our findings highlight that *mcr* has the potential to spread colistin resistance to potentially disparate strains and niches in Qatar, posing a risk that requires intervention.

## 1. Introduction

Colistin (polymyxin E) is a cationic polypeptide antibiotic. Colistin was approved in 1959 to treat Gram-negative bacterial infections but was withdrawn in the 1980s due to its nephrotoxicity and neurotoxicity [1,2]. However, the emergence of multi-drug-resistant (MDR) bacteria and the scarcity of new antibiotics resulted in the reintroduction of colistin in the 2000s as a last-resort antibiotic to treat complicated MDR infections [3,4,5].

Resistance to colistin was associated with chromosomal mutations that lead to lipopolysaccharide modifications [6]. However, in 2015, the plasmid-borne mobile colistin resistance gene (*mcr-1*) was first reported in an *Escherichia coli* isolated from a pig in China [7]. *mcr-1* encodes for a phosphoethanolamine transferase, which modifies lipid A by increasing its positive charge, resulting in resistance to colistin [7]. Since the discovery of *mcr-1*, multiple *mcr* genes and variants have been identified (*mcr-2* to *mcr-10*) [8,9,10,11,12,13,14,15,16]. However, an analysis of 1386 *mcr* carrying Gram-negative bacterial genomes from the NCBI database found that *E. coli* (952 isolates, ≈68.7%) were the most common carriers of *mcr-1* so far [17]. To date, *mcr-1* has been reported in isolates belonging to different species from diverse sources, including animals, humans, and the environment [17,18,19].

The wide and relatively rapid dissemination of *mcr-1* can be attributed to the robust plasmids that carry this gene. Previous studies have reported several *mcr-1*-carrying plasmids with varying sizes (58–256 kb) and Inc types, including IncI2, IncHI2, IncX4, IncP, IncY, IncF, IncFI, IncFII, IncFIB, INcK2, IncN, and IncQ. However, IncI2, IncHI2, and IncX4 appear to be the most common carriers of *mcr-1* [7,18,20,21,22,23,24,25]. Notably, a single *E. coli* isolate can harbor two different *mcr-1*-carrying plasmids simultaneously, increasing the possibility of the transmission of this gene [26]. The mobility of *mcr-1* poses an apparent public health concern associated with the spread of colistin resistance, rendering this critically important antibiotic ineffective. Additionally, the latter is complicated further because *mcr-1*-carrying plasmids often harbor other antimicrobial resistance (AMR) genes, including those encoding resistance to β-lactams, fluoroquinolones, and tetracyclines, among others [20].

Many of the *mcr* genes were first reported in animals, highlighting their importance as a reservoir for colistin resistance [8,9,10,11,12,13,14,15,16,20,27]. It was hypothesized that the dissemination of *mcr-1* among livestock-associated isolates was accelerated by using colistin as a growth promoter and/or prophylaxis in the treatment of livestock [18,19,20]. Therefore, it is important to closely monitor the emergence of *mcr* and associated plasmids and strains in livestock to devise appropriate control strategies and reduce the dissemination of colistin resistance via the food chain and/or environment. We previously reported colistin resistance in samples from humans, broiler chickens, and chicken carcasses in Qatar [28,29,30]. However, our previous analysis focused primarily on detecting *mcr* using PCR analysis. Here, the *mcr-1*-harboring isolates from broiler chickens (fecal matter) and chicken carcasses were subjected to an in-depth investigation to determine (1) the location of *mcr-1* (plasmid-borne or chromosomal), (2) the transmissibility of plasmid-borne *mcr-1*, (3) the stability of existence of *mcr-1* in the isolates after biofilms formation, and (4) the plasmid types and other properties (sequence type, acquired resistome, virulence) of the strains that carried this gene.

## 2. Results

### 2.1. Antibiotic Susceptibility Profiles of the mcr-1-Carrying E.coli

**Figure 1:**
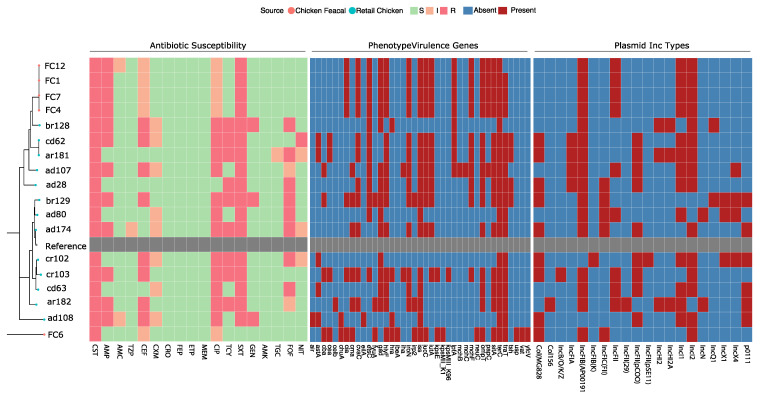
Phylogenetic tree, antimicrobial susceptibility profiles, virulence factors, and plasmid incompatibility (Inc) types of the 18 *mcr-1*-carrying *Escherichia coli* isolated from retail chicken carcasses and broiler chicken fecal samples. *E. coli* K-12 MG1655 (GenBank: CP014225.1) was used as a reference strain for the phylogeny and indicated in grey. The antibiotics tested were ampicillin (AMP), amoxicillin-clavulanic acid (AMC), piperacillin-tazobactam (TZP), cephalothin (CEF), cefuroxime (CXM), ceftriaxone (CRO), cefepime (FEP), ertapenem (ETP), meropenem (MEM), ciprofloxacin (CIP), tetracycline (TCY), sulfamethoxazole-trimethoprim (SXT), gentamicin (GEN), amikacin (AMK), fosfomycin (FOF), nitrofurantoin (NIT), and colistin (CST). S, I, and R correspond to susceptible, intermediate, and resistant in the antibiotic susceptibility profiles, respectively. For the virulence factors and plasmid Inc types, red indicates presence, and blue indicates the absence from the genome assembly.

All 18 isolates were resistant to colistin with MICs ranging between 3 and 12 μg/mL (median = 6 μg/mL; Table 1). The majority of the isolates (17 of 18, ≈94.4%) were identified as MDR due to the resistance to the ≥3 antibiotic classes [30]. However, none of isolates were extended-spectrum β-lactamase or carbapenemase producers because they were sensitive to β-lactam/β-lactamase inhibitor combinations; second-, third-, and fourth-generation cephalosporins; and carbapenemases (Figure 1). Resistances to sulfamethoxazole–trimethoprim, ciprofloxacin, tetracycline, and fosfomycin were highly detected at 94.44%, 66.7%, 50%, and 44%, respectively.

### 2.2. Genomic Features and Diversity

The isolates’ genomic features, including phylogroups, sequence types (ST), antibiotic resistance genes (ARG), plasmid Inc types, FimH type, and virulence genes, are listed in Table 1 and shown in Figure 1. The isolates belonged to 12 STs, with four isolates (22%) belonging to ST 602, two to ST295, and another two to ST48. The remaining isolates belonged to ST10, ST1011, ST155, ST224, ST3270, ST34, ST355, ST6448, and ST744. Lastly, one isolate (ar182) was not assigned an ST because the housekeeping gene alleles did not match any known ST in the database. Notably, the four isolates (FC1, FC4, FC7, and FC12) that belonged to ST 602 were isolated from fecal samples collected from chickens on the same farm. These isolates also had similar ARGs, virulence factors, and plasmid types.

As for phylogroups (Figure 1), most of the isolates (*n* = 9; 50%) belonged to phylogroup B1, followed by phylogroup A (*n* = 7; 38.9%), and an isolate belonged to each of the phylogroups B2 and E, respectively.

The FimH types FimH86 and FimH54 were the most common, with four isolates each. Two isolates had FimH39, and the remaining isolates belonged to individual FimH types. Analysis of the plasmid Inc types showed that all isolates carried at least four plasmids and at most nine (median = 6.5).

### 2.3. Location of the mcr-1

The location of the *mcr-1* was analyzed using mlplasmids and MGEfinder (Table 2) [31,32]. The mlplasmids showed that most isolates (83.3%) carried *mcr-1* on a plasmid. However, analysis with MGEfinder showed that only 66.67% of the isolates harbored the gene on a plasmid. The discrepancies were in three cases where mlplasmids flagged the gene to be on plasmid, while MGEfinder assigned it as chromosomal. In another case, the opposite occurred between the two programs. However, there was a concordance of 83.3%. Moreover, the results of the MGEfinder analysis showed that all isolates that had plasmid-harbored *mcr-1* carried the gene on an IncI2 plasmid, except for two isolates that carried *mcr-1* on IncX4.

**Figure 2:**
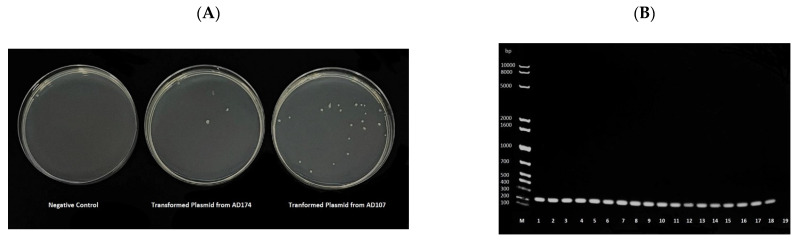
(**A**) Representative image of the One Shot^TM^ OmniMAXTM 2 T1R *E. coli* growth in Muller–Hinton media supplemented with 4 μg/mL of colistin following heat-shock transformation. The negative control underwent the heat-shock method without adding plasmid. (**B**) Agarose gel electrophoresis of the *mcr-1* PCR product from DNA extracts of the transformants. The first well, labelled M, contained a 1 kb Ladder. Plasmids extracted from each sample were transformed into a chemically competent *E. coli.* The origin of the DNA in each well were as follows, 1: ad28, 2: ad80, 3: ad107, 4: ad108, 5: ad174, 6: ar181, 7: ar182, 8: br128, 9: br129, 10: cd62, 11: cd63, 12: cr102, 13: cr103, 14: FC1, 15: FC4, 16: FC6, 17: FC7, 18: FC12, 19: PCR negative control.

Plasmids were extracted from each isolate and transformed into a chemically competent colistin-sensitive *E. coli* to verify the in silico results and assess the transmissibility of the *mcr-1*. Successful transformation was evaluated by colonies growing on colistin-supplemented media, and the transformation resulted in colonies in all cases (Figure 2). All transformants were colistin-resistant, confirming that *mcr-1* was carried on plasmids in all the isolates. DNA was extracted from the colistin-resistant transformants and by *mcr-1*-specific PCR analysis for further verification. All the transformants showed positive amplification of *mcr-1* (Figure 2).

### 2.4. Trasnmissibility of the mcr-1 via Conjugation

**Figure 3:**
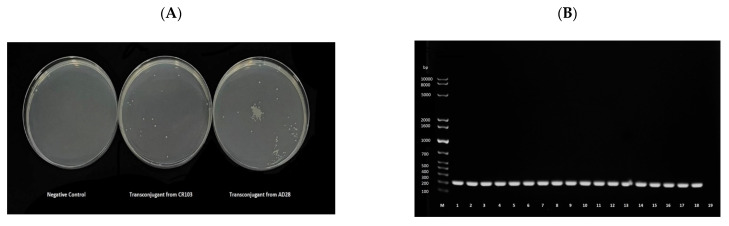
(**A**) Representative image of the trans-conjugant *E. coli* K12 growth in Muller–Hinton media supplemented with 2 μg/mL of colistin and 2000 μg/mL of streptomycin following conjugation. The negative control underwent the conjugation method without adding donor cells. (**B**) Agarose gel electrophoresis of the *mcr-1* PCR product from DNA extracts of the transconjugants. The first well, labeled M, contains a 1 kb Ladder. mcr-1 positive *E. coli* isolates (donors) underwent conjugation with *E. coli* K12, strain IM93b (recipient). The origin of the DNA in each well were as follows, 1: ad28, 2: ad80, 3: ad107, 4: ad108, 5: ad174, 6: ar181, 7: ar182, 8: br128, 9: br129, 10: cd62, 11: cd63, 12: cr102, 13: cr103, 14: FC1, 15: FC4, 16: FC6, 17: FC7, 18: FC12, 19: PCR negative control.

To further assess the transmissibility of *mcr-*carrying plasmids, conjugation experiments were performed. Transconjugants were obtained for all donors. Hence, conjugation was successful in all cases (Figure 3). For confirmation, DNA was extracted from the transconjugants that grew on MH media containing colistin and streptomycin and subjected to *mcr-1-*specific PCR analysis. Transconjugants from all donors showed positive amplification of *mcr-1* (Figure 3).

### 2.5. Presistence of the mcr-1 Plasmids in Biofilms

**Figure 4:**
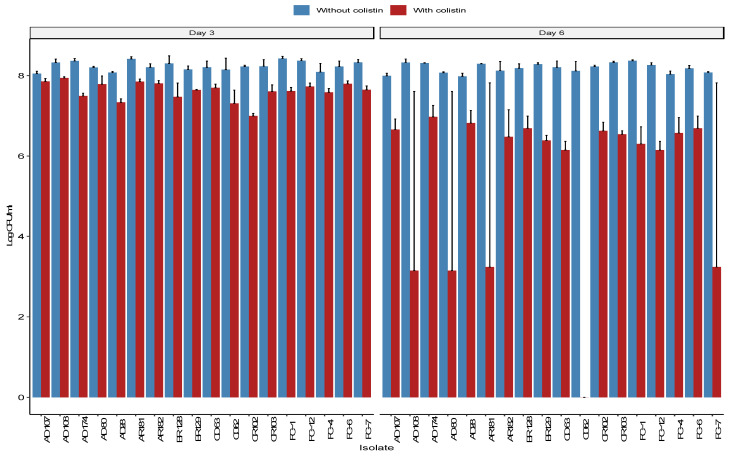
Log bacterial concentration (CFU/mL) was obtained from each sample on days 3 and 6 of the biofilm formation. Each count was performed on two media, one in Mueller–Hinton (MH) agar (blue) and one in MH supplemented with 4 μg/mL of colistin (red). The left side shows the bacterial concentration on day 3, and the right side represents the bacteria concentrations on day 6.

All isolates showed an ability to form biofilms as measured by the absorbance at 570 nm for the crystal violet dye (see Figure S1a,b). A Wilcoxon signed-rank test indicated that the log CFU/mL from the biofilms was significantly lower on day 6 compared to day 3 on both MH agar without colistin (*p* = 0.033) and MH with colistin (*p* = 7.6 × 10^−6^) (Figure 4). However, the reduction in the colistin-supplemented medium was significantly larger than the reduction in the non-supplemented medium (*p* = 7.6 × 10^−6^; 0.055 log CFU/mL vs. 2.19 log CFU/mL), suggesting a partial loss of *mcr-1*-carrying plasmids in a portion of the biofilm *E. coli* population.

## 3. Discussion

Several studies have shown that the prevalence of *mcr*-carrying colistin-resistant *E. coli* is usually higher in livestock than in humans [33]. For example, studies in Vietnam found that 97.2% of the domestic chickens tested carried *mcr-1*-positive *E. coli* with evidence of clonal relationships [34]. Similarly, in Lebanon, approximately 98% of the colistin-resistant *E. coli* retrieved from feces of broiler chicken were *mcr-1*-positive [35]. Additionally, there are increasing reports of acquiring *mcr* by strains that carry other antibiotic resistance determinates such as ESBL- or pAMP-C-encoding genes [36,37]. These observations and the mobility of *mcr* highlight the importance of monitoring and analyzing the epidemiology and transmissibility of *mcr* in livestock and associated products in different countries. This is imperative to reduce the risk of spreading colistin resistance via animal farming, the food chain, and the environment, as well as to control potentially complicated infections. In previous studies, 270 *E. coli* from retail chicken carcasses and 172 from fecal samples from broiler chicken farms were isolated in Qatar [28,38]. In those studies, the prevalence of *mcr-1*-carrying *E. coli* was 31.9% in retail chicken carcasses and 15.6% in the feces of broiler chickens. However, the isolates were not investigated further. On the basis of these observations and studies, 18 *mcr-1*-positive chicken-associated *E. coli* were randomly selected (13 from retail chicken and 5 from broiler chicken) for an in-depth analysis to understand further the emergence and potential spread of *mcr* in Qatar.

Most of the isolates included in this study showed an MDR phenotype. However, none of the isolates were likely ESBL producers as none of them were resistant to third- or fourth-generation cephalosporins, and they were not carbapenem resistant. Interestingly, the majority of the isolates were ciprofloxacin resistant. Ciprofloxacin and colistin are frequently used as growth promoters and prophylactic agents [39]. The latter might have contributed to the observed resistance in these isolates. However, data on antibiotic use in these animals were not available. Regardless, the over-reliance on antibiotics in animal farming might drive the selection of more resistant strains that can pose a risk for both animals and humans.

Phenotypic and genomic analyses revealed a notable diversity between the isolates (Figure 1 and Table 1). Isolates belonging to Phylogroup B1 were the most common (Table 1). This was expected because a high proportion of group B1 is common in mammals and birds [40,41]. One fecal isolate (FC6) belonged to phylogroup B2, common in humans and associated with inflammatory bowel disease and urinary tract infections [41,42]. However, the most frequently detected STs were ST602 (22%), while the remaining isolates were mainly assigned disparate STs. All four isolates that belonged to ST602 were closely related and were obtained from feces of broiler chickens on the same farm, highlighting the possibility of clonal transmission. These four isolates carried identical ARGs, virulence genes, and plasmid replicons. Notably, ST602 is associated with the global spread of AMR in humans and food-producing animals, particularly poultry [43]. However, detecting different and diverse STs is consistent with other studies that found that the *mcr-1*-positive *E. coli* were not necessarily clonal [44,45].

Each *mcr-1*-carrying *E. coli* isolate in this study carried between 4 and 9 plasmid Inc types (median = 6.5). The majority of the isolates (*n* = 10) harbored the IncI2 plasmid, one of the most prevalent *mcr-*carrying plasmids detected in poultry, human, and environmental samples [33]. The bioinformatics analysis also revealed that two isolates had *mcr-1* on IncX4 plasmids (Table 2), which are also associated with the global dissemination of *mcr*-mediated colistin resistance [46]. The *mcr* in the remaining isolates was not assigned to a plasmid Inc type and had a low probability of being on a plasmid according to in silico analysis. However, there were discrepancies between the two algorithms used to identify the location of *mcr-1*. This discrepancy might have resulted from limitations associated with short-read WGS for plasmids or because the flanking regions of the *mcr-1* did not match a known plasmid sequence. The latter is very interesting because *mcr-1* in all the isolates was plasmid-born using heat-shock and conjugation assays, suggesting the potential to detect unknown *mcr-1*-carrying plasmid types. To date, more than 12 *mcr-1*-carrying plasmid types have been identified [26]. This plasmid diversity contributes to *mcr-1* spread; however, the exact role and diversity of the plasmids still require further investigation.

Whether the gene is carried on a chromosome or a plasmid might result in different transmission dynamics. The *mcr-1* is typically flanked by IS*Apl1* transposable elements [47]. IS*Apl1* is thought to be the element that mobilizes the gene on plasmids and allows for it to be integrated into chromosomes [44,47]. A gene on a chromosome can be transmitted vertically within a clonal lineage, while carrying it on a plasmid (in addition to mobilization through IS*Apl1*) will enable it to be transferred horizontally between clones and to other bacterial genera. Therefore, a transformation experiment was performed to validate in silico analysis. The transformation was successful for plasmids extracted from all the isolates, as evidenced by growth on colistin-supplemented media and subsequent detection of *mcr-1* in the transformants (Figure 2).

Similarly, mating in experiments was conducted to evaluate further if *mcr*-*1* was transmissible plasmid borne. Conjugation was observed with all 18 isolates as donors, as evidenced by the growth of transconjugants on media supplemented with colistin and streptomycin and subsequent detection of *mcr-1* in the transconjugants (Figure 3). The natural transformation has been observed in *E. coli*, albeit at low levels and seemingly environmentally dependent [48,49,50]. For example, a 1996 study that assessed natural transformation in *E. coli* in environmental water samples showed that the transformation efficiency was higher in samples taken downstream of wastewater effluent than elsewhere [48]. Additionally, conjugation was formerly shown for IncI2 and IncX plasmids [5,7,13,51]. Regardless, the data in this study indicate that *mcr* is transmissible and, as such, poses a problem that requires immediate action to identify and limit the factors that are driving the emergence of *mcr* in livestock and associated products in Qatar. Further investigations are necessary to examine the frequency of conjugation and whether it leads to similar resistance levels in the recipient strain and to track *mcr-1*-carrying plasmid transmission to other niches and bacterial hosts.

AMR dissemination continues to be a significant public health challenge and has been receiving increased research interest. However, ARGs are not the only mechanism of resistance/persistence possessed by bacteria. Biofilm formation has been shown to increase antimicrobial tolerance and resistance and promote ARG transfer [52]. However, dogmatically *mcr-1*-carrying plasmids might be unstable and can incur a high fitness cost [53]. It was hypothesized that when a more cost-efficient form of resistance exists, the bacteria will shed high-cost plasmids. However, in reality, this remains not well investigated for *mcr-1*-carrying plasmids. Therefore, a biofilm formation assay was performed to test this hypothesis. There was a significant reduction in biofilm-associated bacterial density between days 3 and 6 on MH agar without and with colistin (*p* = 0.033 and *p* = 7.6 × 10^−6^, respectively). The reduction in bacterial density on day 6 was incompatible with the results of the crystal violet assay. The discrepancy was likely due to the differences between the two methods. CFU plate counts is a direct method and accounts only for viable cells, while the crystal violet assay is indirect and does not distinguish between viable and non-viable cells. The reduction in bacterial CFU on the MH media was likely due to the bacteria reaching a growth plateau and resource limitation. However, the decline in colistin resistance was significantly higher than the reduction in bacterial CFU (*p* = 7.6 × 10^−6^; 0.055 log CFU/mL vs. 2.19 log CFU/mL), indicating that *mcr-1*-carrying *E. coli* persistence in biofilms is reduced after six days. This was likely due to a combination of factors, including energy conservation, protection in biofilms (tolerance/resistance to stressors such as antibiotics), and resource limitations. The results are consistent with experiments performed with *Klebsiella pneumoniae* using an *mcr-1* recombinant plasmid and with the IncX4 plasmid in *E. coli* [46,53]. The majority of the isolates in this study carried the gene on IncI2 plasmids, indicating that a similar fitness cost existed for these plasmids. Despite the reduction in resistant CFUs, it should be noted that a significant number of resistant and *mcr-1*-carrying bacteria persisted in the 6-day-old biofilms. This might increase the potential of *mcr-1* transmission, even under unfavorable survival conditions. Further studies are required to assess the duration of *mcr* persistence and its transmissibility in single- and multi-species biofilms, respectively.

## 4. Materials and Methods

### 4.1. Bacterial Isolates

The *E. coli* strains investigated in this study were isolated between September 2016 and April 2018 and are described in previously published studies [28,38]. Briefly, *E. coli* were isolated from chicken carcasses collected from three major hypermarkets in Qatar and from chicken fecal samples from multiple broiler farms. The isolates were collected using a stratified random sampling approach under the supervision of the Ministry of Public Health (MoPH) and the Ministry of Municipality (MM). All the isolates were stored at −80 °C. Eighteen isolates that tested positive for *mcr-1* by PCR were randomly selected for this study.

### 4.2. Antimicrobial Susceptibility Testing

The susceptibility of the *mcr-1*-positive *E. coli* to 17 antibiotics was assessed using E-test strips. Briefly, each isolate was grown on blood agar plates (BD-Medysinal FZCO, Dubai, UAE) overnight. Colonies from each plate were suspended in a phosphate buffer solution to achieve a 0.5 McFarland (McF) as measured by DensiCHEK Plus (bioMérieux, Marciy-L’Étoile, France), and the suspension was then spread onto Mueller–Hinton (MH) agar plates (HiMedia, Maharashtra, India). The E-test strips for each antibiotic (Liofilchem, Roseto delgi Abruzzi, Italy) were applied to the agar surface, and the plates were incubated at 37 °C overnight. The antibiotics tested were ampicillin (AMP), amoxicillin-clavulanic acid (AMC), piperacillin-tazobactam (TZP), cephalothin (KF), cefuroxime (CXM), ceftriaxone (CRO), cefepime (FEP), ertapenem (ETP), meropenem (MEM), ciprofloxacin (CIP), tetracycline (TCY), sulfamethoxazole-trimethoprim (SXT), gentamicin (GEN), amikacin (AMK), fosfomycin (FOF), nitrofurantoin (NIT), and colistin (CST). The minimum inhibitory concentrations (MICs) were interpreted following the Clinical and Laboratory Standards Institute (CLSI) [54]. *E. coli* strains ATCC 25922 and ATCC 35218 were used as controls.

### 4.3. Whole-Genome Sequencing

Genomic DNA was extracted from the isolates using the QIAamp^®^ UCP Pathogen mini kit (Qiagen, Hilden, Germany) and following the manufacturer’s protocol. The DNA was then quantified with the Qubit dsDNA high-sensitivity assay (Thermo Fisher, Waltham, MA, USA). Whole-genome sequencing was performed on the BIGSEQ-500 (Beijing Genomics Institute, Shenzhen, China). Briefly, the genomic DNA was randomly fragmented with the Covaris instrument (Covaris LLC., Woburn, MA, USA), and 200–400 bp fragments were selected using the Agencourt AMPure XP beads (Beckman Coulter, Brea, CA, USA). The fragments were then end-repaired and $3^{\prime }$ adenylated, and adaptors were ligated. PCR was then performed to amplify the fragments, which were purified using the Agencourt AMPure XP beads. Lastly, the DNA was circularized and then sequenced using DNA nano-balls (DNB), resulting in 150 bp paired reads.

### 4.4. Bioinformatics Analysis

The quality of the raw reads was assessed using Fastqc (https://www.bioinformatics.babraham.ac.uk/projects/fastqc/, accessed on 1 November 2021), and trimming was performed using TrimGalore (https://www.bioinformatics.babraham.ac.uk/projects/trim_galore/, accessed on 1 November 2021) to remove adaptors and low-quality reads. Contamination was checked using kraken2 v2.1.1 [55]. The trimmed reads were assembled using SPAdes v3.13.0 and optimized with Unicycler v0.4.9 [56,57]. The final assemblies (*n* = 18) are available on the NCBI website under BioProject PRJNA786273 (The genome accessions are listed in Table S1).

Core genome alignment of the isolates with *E. coli* K-12 MG1655 (GenBank accession: CP014225.1) as the reference strain was performed using the RedDog read mapping pipeline (https://github.com/katholt/reddog-nf, accessed on 1 November 2021). The maximum-likelihood phylogenetic tree was constructed using FastTree [58,59]. Phylotyping was performed using ClermonTyping v20.03 [60]. Multi-locus sequence typing (MLST) and antimicrobial resistance gene (ARG) identification were performed using SRST2 v0.2.0 [61]. The incompatibility (Inc) types of the plasmids carried by the isolates were identified using PlasmidFinder v2.1.6 [62]. Virulence genes were identified with VirulenceFinder v2.0, and the FimH types were determined with FimTyper v1.0 [63,64]. Lastly, the locations (chromosomal or on a plasmid) of the *mcr-1* in each isolate were inferred using mlplasmids v1.0.0 and MGEfinder v1.0.3 [31,32].

### 4.5. Plasmid Extraction and Screening for mcr-1 Using PCR Analysis

The 18 colistin-resistant isolates were sub-cultured in Luria–Bertani (LB) broth and grown overnight (Merck, Dramstadt, Germany). Then, plasmid DNA was extracted using the QIAprep^®^ spin miniprep kit (Qiagen, Hilden, Germany) as described in the manufacturer’s instructions. The concentration and purity of the eluted plasmid DNA were determined using the NanoDrop™ 2000c (Thermo Fisher Scientific, Waltham, MA, USA).

The presence of *mcr-1* in the plasmid extracts was confirmed with PCR. The PCR reaction contained 0.5 μM of each primer (MCR1_22697 F1: cacttatggcacggtctatga, MCR1_22810 R1: cccaaaccaatgatacgcat) [65], 30 ng of DNA, 12.5 μL of A PCR master mix (Hot star Taq Plus, Qiagen, Hilden, Germany), 1x of gel loading dye (CoralLoad, Qiagen, Hilden, Germany), and DEPC-treated water up to a volume of 25 μL. The reactions were amplified in a Thermal Cycler using the following program: denaturation at 95 °C for 15 min; 25 cycles of 95 °C for 30 min, 58 °C for 90 min, and 72 °C for 1 min; and a final extension at 72 °C for 10 min. The amplified PCR products were subjected to electrophoresis in a 1.2% agarose gel (AgaroseLE, Ambion^®^, Austin, TX, USA) stained with ethidium bromide (Promega, Madison, WI, USA). The gel was visualized using a Molecular Imager^®^ Gel Doc™ XR System 170–8170 (Bio-rad, Hercules, CA, USA).

### 4.6. Assessing mcr-1 Transmissibility Using the Heat Shock Method

The extracted plasmids were transformed into One shot™ OmniMAX™ 2 T1^R^ chemically competent *E. coli* cells (Thermo Fisher Scientific, Waltham, MA, USA) by heat shock [66,67]. Briefly, 50 μL of the chemically competent *E. coli* were mixed with 3 μL of the extracted plasmid and incubated on ice for 1 h. The mixture was then heat-shocked by placing it in a 42 °C water bath for 45 s, which was followed by incubation on ice for 2 min. After heat shock, 1 mL of freshly prepared LB broth was added, and the mixture was incubated at 37 °C in a shaking incubator at 200 rpm for 1 h. The mixture was centrifuged at 3000 rpm for 5 min, and 0.9 mL of the supernatant was removed. The pellet was resuspended in the remaining solution. Moreover, transformants were selected by spreading the suspension on MH agar plates supplemented with 4 μg/mL of colistin. Transformant colonies were subcultured in LB broth overnight and subjected to plasmid extraction and *mcr-1* PCR analysis to confirm the transmission of *mcr-1*-carrying plasmids. Chemically competent *E. coli* with 3 μL sterile deionized water were used as a negative control.

### 4.7. Assessing mcr-1 Transmissibility via Conjugation Assays

Conjugation experiments were conducted to assess further the transmissibility of the *mcr-1*-carrying plasmids. The *mcr-1*-positive isolates were used as donors, and the streptomycin-resistant *E. coli* K-12 strain IM93B (BEI Resources, Manaas, VA, USA) as the recipient. Briefly, all isolates were inoculated into 4 mL of LB broth and incubated for 5 h at 37 °C with shaking at 200 rpm. The donor strain was then mixed with the recipient strain at a proportion of 1:3 and incubated for 5 h at 37 °C in a shaking incubator at 200 rpm. The mixture was spread on MH plates containing 2000 μg/mL streptomycin and 2 μg/mL colistin and incubated at 37 °C for 24 h to select transconjugants. DNA was extracted from the transconjugants and screened by PCR analysis for *mcr-1* as described above to verify that the conjugation occurred. As a control, the recipient and donor were separately plated on a streptomycin plus colistin plate.

### 4.8. Assessing the Persistence of mcr-1 Plasmids in Biofilms

The persistence of *mcr-1* plasmids in the biofilm was assessed using the crystal violet biofilm assay as described by Hassan et al. (2020) [46]. Briefly, *mcr-1*-positive *E. coli* were grown in LB broth. The cultures were diluted 100-fold and incubated at 37 °C for 2 h in a shaking incubator at 200 rpm. The optical density at 600 nm (OD_600_) was adjusted to 0.05. Next, 2 mL aliquots of the cultures were transferred to 5 mL sterile borosilicate glass vials. One set of duplicate vials for each isolate was incubated for 3 days and another for 6 days in an incubator at 37 °C. Then, non-adherent bacterial cells were removed by washing with 2 mL of sterile distilled water. The experiment was performed twice, once to assess the biofilm formation and the second to assess the persistence of *mcr-1-*carrying plasmids.

To assess biofilm formation, the glass vials were air-dried after washing the non-adherent cells and stained with 2 mL of crystal violet solution (Anamol Laboratories Pvt Ltd., Maharashatra, India) for 15 min at room temperature. The vials were then washed with 2 mL sterile distilled water several times and dried to remove the excess crystal violet. Then, 2 mL of acetic acid (Fisher Scientific, Waltham, MA, USA) was added to each vial, and they were incubated for 1 h at room temperature. The optical density of the suspension was measured at 570 nm using a LabQuest spectrophotometer (Vernier, Beaverton, OR, USA). Vials containing only LB broth were used as negative controls.

To assess the *mcr-1-*carrying plasmids, the biofilms were resuspended in 1 mL of LB broth after removing the non-adherent bacterial then serially diluted components (10-fold). Next, 100 μL of each dilution was spread on MH agar plates without and with 4 μg/mL of colistin. The plates were incubated at 37 °C overnight, and colony-forming units (CFU) were counted.

### 4.9. Data Analysis

Statistical analyses were performed using R v4.1.0 [68]. Figures were generated with ggplot v3.3.5, ggpubr v0.4.0, ggtree v3.0.4, and aplot v0.1.1 [69,70,71]. The differences in growth in biofilm assays were assessed using a Wilcoxon signed-rank test as the data did not follow a normal distribution.

## 5. Conclusions

Antibiotic use in humans and animals facilitates AMR emergence and dissemination and the interactions between humans, animals, and the environment. Consequently, the One Health approach is instrumental in tackling AMR, locally and globally. Colistin resistance, particularly *mcr-1-*mediated resistance, has been reported in different hosts and niches and constitutes a prime target for One-Health-based intervention. A few studies have previously reported *mcr-1-*mediated colistin resistance in Qatar’s humans, animals, and food products. In this study, mcr-1-carrying *E. coli* were subjected to a rigorous analysis that revealed the *mcr-1* (1) was plasmid-borne and transmissible, (2) occurred in diverse genetic backgrounds along with other important ARGs, (3) persists in biofilms at a cost, and (4) can pose a significant problem if not tackled appropriately. Consequently, there is a need to continue investigations and identify and monitor the factors that drive the emergence and spread of *mcr* in Qatar. This will be critical to developing a suite of interventions under a One Health approach.

## Figures and Tables

**Table 1:** Colistin minimum inhibitory concentrations (MICs), genomic analysis, and properties of the *mcr-1*-harbouring *E. coli* isolates.

Isolate	MIC (μg/mL)	Source	Phylogroup	Sequence Type	FimH Type	Plasmid Inc Types	Virulence Genes	Other Antibiotic Resistance Genes
ad107	8	Retail chicken	B1	224	*fimH39*	IncFIA, IncFIB(APNANA1918), IncFII, IncFII(pCoo), IncI1, IncI2, IncX4	*cba*, *cma*, *etsC*, *gad*, *hlyF*, *iha*, *iroN*, *iss*, *iucC*, *iutA*, *lpfA*, *mchB*, *mchC*, *mchF*, *ompT*, *ompT*, *papC*, *sitA*, *terC*, *traT*	*aadA2_104*, *aph3″-Ib_146*, *aph6-Id.v2_172*, *AmpC1_286*, *cmlA1_2259*, *dfrA14.v1_2362*, *fosA4_1904*, *mphA_2174*, *mphB.v2_2176*, *strA.v1_2409*, *strB.v1_2410*, *sul3_2285*, *TEM-206_1678*
ad108	12	Retail chicken	E	1011	*fimH31*	Col(MG828), IncFII(pCoo), IncI1, IncI2, pNA111	*air*, *astA*, *chuA*, *eilA*, *hra*, *iss*, *terC*, *traT*	*aac3-IId_16*, *aadA8b_121*, *aph3″-Ib_146*, *aph3-Ia.v1_165*, *aph6-Id.v2_172*, *AmpC1_286*, *catA1_2229*, *dfrA15.v2_2365*, *erm42_2125*, *floR.v1_2271*, *fosA4_1904*, *mphA_2174*, *mphB.v2_2176*, *qnrS1_2006*, *strA.v1_2409*, *strB.v1_2410*, *sul3_2285*, *TEM-135.v2_1673*, *tetA.v1_2317*
ad174	8	Retail chicken	A	10	*fimH54*	Col(MG828), IncFIB(APNANA1918), IncFIC(FII), IncFII(pCoo), IncI2, pNA111	*cma*, *cvaC*, *gad*, *hlyF*, *iroN*, *iss*, *iucC*, *iutA*, *ompT*, *sitA*, *terC*, *traT*	*aadA8b_121*, *aph3″-Ib_146*, *aph3-Ia.v1_165*, *aph6-Id.v1_171*, *AmpC1_286*, *dfrA12_2360*, *fosA4_1904*, *mphA_2174*, *mphB.v2_2176*, *strA.v1_2409*, *strB.v1_2410*, *sul1_2283*
ad28	6	Retail chicken	B1	6448	*fimH60*	Col(MG828), IncFIA, IncFIB(APNANA1918), IncFIC(FII), IncFII(pCoo), IncI1, IncI2	*cvaC*, *etsC*, *gad*, *hlyF*, *iroN*, *iss*, *iucC*, *iutA*, *mchF*, *ompT*, *sitA*, *terC*, *traT*, *tsh*	*erm42_2125*, *floR.v1_2271*, *fosA4_1904*, *mphA_2174*, *mphB.v2_2176*, *tetA.v1_2317*
ad80	12	Retail chicken	A	34 *	*fimH24*	IncFIB(APNANA1918), IncFIC(FII), IncFII, IncI1, IncN, IncX1, IncX4	*etsC*, *gad*, *iss*, *terC*, *traT*	*aac3-IV_21*, *aadA24_108*, *aph3-Ia.v1_165*, *aph4-Ia_166*, *AmpC1_286*, *cmlA1_2259*, *floR.v1_2271*, *mefB_2098*, *mphA_2174*, *mphB.v2_2176*, *qnrB7_1992*, *sul3_2285*, *TEM-128.v2_1671*
ar181	8	Retail chicken	B1	295	*fimH38*	Col(MG828), IncFIA, IncFIB(APNANA1918), IncFII(pCoo), IncHI2, IncHI2A, IncI1, IncI2	*astA*, *cea*, *cvaC*, *etsC*, *hlyF*, *iroN*, *iss*, *iucC*, *iutA*, *lpfA*, *lpfA*, *mchF*, *ompT*, *sitA*, *terC*, *traT*, *tsh*	*aadA15_101*, *AmpC1_286*, *dfrA1.v1_2357*, *fosA4_1904*, *mphA_2174*, *mphB.v2_2176*, *qnrB7_1992*, *sat-2_193*, *tetA.v1_2317*
ar182	8	Retail chicken	A	NF	*fimH54*	Col156, IncFIB(APNANA1918), IncFII, IncFII(29), IncHI2, IncHI2A, IncI1, IncN, pNA111	*celb*, *cma*, *cvaC*, *fyuA*, *gad*, *hlyF*, *iroN*, *irp2*, *iss*, *ompT*, *sitA*, *terC*, *traT*	*aadA24_108*, *aph3″-Ib_146*, *aph3-Ia.v1_165*, *aph6-Id.v1_171*, *AmpC1_286*, *cmlA1_2259*, *dfrA14.v1_2362*, *floR.v1_2271*, *mphA_2174*, *mphB.v2_2176*, *strA.v1_2409*, *strB.v1_2410*, *sul3_2285*, *TEM-207_1756*, *tetA.v1_2317*
br128	6	Retail chicken	B1	155	*fimH121*	IncFIB(APNANA1918), IncHI2, IncHI2A, IncI2, IncQ1	*etsC*, *gad*, *hra*, *lpfA*, *terC*	*aac3-IIa.v1_12*, *aadA2_104*, *aph3″-Ib_146*, *aph3-Ia.v1_165*, *aph6-Id.v1_171*, *AmpC1_286*, *catA1_2229*, *cmlA1_2259*, *dfrA12_2360*, *floR.v1_2271*, *mphA_2174*, *mphB.v2_2176*, *strA.v1_2409*, *strB.v1_2410*, *sul3_2285*, *TEM-207_1756*, *tetA.v1_2317*
br129	6	Retail chicken	A	744	*fimH54*	Col(MG828), IncFIB(APNANA1918), IncFIC(FII), IncQ1, IncX1, IncX4, pNA111	*cba*, *cia*, *cma*, *cvaC*, *etsC*, *fyuA*, *gad*, *hlyF*, *iroN*, *irp2*, *iss*, *iucC*, *iutA*, *mchF*, *ompT*, *sitA*, *terC*, *traT*, *tsh*	*aadA5_114*, *aph3″-Ib_146*, *aph3-Ia.v1_165*, *aph6-Id.v1_171*, *AmpC1_286*, *catA1_2229*, *dfrA17_2369*, *floR.v1_2271*, *mphA_2174*, *mphB.v2_2176*, *strA.v1_2409*, *strB.v1_2410*, *sul1_2283*, *TEM-206_1678*, *tetB.v2_2325*
cd62	12	Retail chicken	B1	295	*fimH38*	Col(MG828), IncFIA, IncFIB(APNANA1918), IncFII(pCoo), IncI1, IncI2	*astA*, *cea*, *cvaC*, *etsC*, *hlyF*, *iroN*, *iss*, *iucC*, *iutA*, *lpfA*, *lpfA*, *mchF*, *ompT*, *sitA*, *terC*, *traT*, *tsh*	*aadA12_98*, *AmpC1_286*, *dfrA1.v1_2357*, *fosA4_1904*, *mphA_2174*, *mphB.v2_2176*, *sat-2_193*
cd63	6	Retail chicken	A	48	*fimH54*	IncFIB(APNANA1918), IncFII, IncFII(pCoo), IncI1, IncI2, pNA111	*astA*, *gad*, *hlyF*, *iroN*, *iss*, *ompT*, *sitA*, *terC*, *traT*	*aadA2_104*, *aph3″-Ib_146*, *aph3-Ia.v1_165*, *aph6-Id.v2_172*, *AmpC1_286*, *cmlA1_2259*, *dfrA14.v1_2362*, *erm42_2125*, *floR.v1_2271*, *fosA4_1904*, *mphA_2174*, *mphB.v2_2176*, *strA.v1_2409*, *strB.v1_2410*, *sul3_2285*, *TEM-206_1678*, *tetA.v1_2317*
cr102	8	Retail chicken	A	48	*fimH41*	Col(MG828), IncFIB(K), IncFII(pCoo), IncFII(pSE11), IncI1, IncI2, IncX1, IncX4, pNA111	*astA*, *gad*, *terC*, *traT*	*aadA_93*, *aph3-Ia.v1_165*, *AmpC1_286*, *dfrA1.v1_2357*, *erm42_2125*, *floR.v1_2271*, *fosA4_1904*, *mphA_2174*, *mphB.v2_2176*, *sat-2_193*, *sul3_2285*, *TEM-176_1646*, *tetA.v1_2317*
cr103	8	Retail chicken	A	3270	*fimH31-like*	Col(MG828), IncB/O/K/Z, IncFIB(APNANA1918), IncFII, IncFII(pCoo), IncI2	*cba*, *cea*, *cia*, *cma*, *cvaC*, *etsC*, *gad*, *hlyF*, *hra*, *iha*, *iroN*, *iss*, *iutA*, *kpsE*, *kpsMIII_K96*, *mchF*, *ompT*, *sitA*, *terC*, *traT*	*AmpC1_286*, *dfrA5_2387*, *fosA4_1904*, *mphA_2174*, *mphB.v2_2176*, *tetA.v1_2317*
FC1	3	Fecal	B1	602	*fimH86*	IncFIB(APNANA1918), IncFII, IncI1, IncI2	*cia*, *cvaC*, *etsC*, *gad*, *hlyF*, *iroN*, *iss*, *iucC*, *iutA*, *lpfA*, *mchF*, *ompT*, *papC*, *sitA*, *terC*, *traT*	*aadA_93*, *aph3″-Ib_146*, *aph6-Id.v2_172*, *AmpC1_286*, *dfrA1.v1_2357*, *mphB.v2_2176*, *sat-2_193*, *strA.v1_2409*, *strB.v1_2410*, *sul2_2284*, *TEM-206_1678*, *tetB.v2_2325*
FC12	3	Fecal	B1	602	*fimH86*	IncFIB(APNANA1918), IncFII, IncI1, IncI2	*cia*, *cvaC*, *etsC*, *gad*, *hlyF*, *iroN*, *iss*, *iucC*, *iutA*, *lpfA*, *mchF*, *ompT*, *papC*, *sitA*, *terC*, *traT*	*aadA_93*, *aph3″-Ib_146*, *aph6-Id.v2_172*, *AmpC1_286*, *dfrA1.v1_2357*, *mphB.v2_2176*, *sat-2_193*, *strA.v1_2409*, *strB.v1_2410*, *sul2_2284*, *TEM-206_1678*, *tetB.v2_2325*
FC4	3	Fecal	B1	602	*fimH86*	IncFIB(APNANA1918), IncFII, IncI1, IncI2	*cia*, *cvaC*, *etsC*, *gad*, *hlyF*, *iroN*, *iss*, *iucC*, *iutA*, *lpfA*, *mchF*, *ompT*, *papC*, *sitA*, *terC*, *traT*	*aadA_93*, *aph3″-Ib_146*, *aph6-Id.v2_172*, *AmpC1_286*, *dfrA1.v1_2357*, *mphB.v2_2176*, *sat-2_193*, *strA.v1_2409*, *strB.v1_2410*, *sul2_2284*, *TEM-207_1756*, *tetB.v2_2325*
FC6	3	Fecal	B2	355	*fimH154*	Col(MG828), IncFIB(APNANA1918), IncFIC(FII), IncI2	*cba*, *cea*, *chuA*, *cia*, *cma*, *fyuA*, *hlyF*, *hra*, *ibeA*, *irp2*, *iss*, *iucC*, *kpsE*, *kpsMII_K1*, *neuC*, *ompT*, *sitA*, *terC*, *traT*, *usp*, *vat*, *yfcV*	-
FC7	3	Fecal	B1	602	*fimH86*	IncFIB(APNANA1918), IncFIIIncI1, IncI2	*cia*, *cvaC*, *etsC*, *gad*, *hlyF*, *iroN*, *iss*, *iucC*, *iutA*, *lpfA*, *mchF*, *ompT*, *ompT*, *papC*, *sitA*, *terC*, *traT*	*aadA_93*, *aph3″-Ib_146*, *aph6-Id.v2_172*, *AmpC1_286*, *dfrA1.v1_2357*, *mphB.v2_2176*, *sat-2_193*, *strA.v1_2409*, *strB.v1_2410*, *sul2_2284*, *TEM-76_1698*, *tetB.v2_2325*

**Table 2:** Results of the in silico analysis of the mcr-1 gene location.

Isolate	Mlplasmids * Probability	MGEfinder * Result
ad107	0.34	chromosome
ad108	0.76	Incl2
ad174	0.76	Incl2
ad28	0.79	Incl2
ad80	0.79	IncX4
ar181	0.62	chromosome
ar182	0.39	chromosome
br128	0.13	chromosome
br129	0.78	IncX4
cd62	0.63	chromosome
cd63	0.71	Incl2
cr102	0.81	chromosome
cr103	0.77	Incl2
FC1	0.76	Incl2
FC12	0.75	Incl2
FC4	0.76	Incl2
FC6	0.76	Incl2
FC7	0.76	Incl2

* Mlplasmids and MGEfinder are bioinformatic tools that statistically classify contigs or genes as chromosmal or plasmid. Mlplasmids provides the porbability that the contig is on a plasmid, while MGEfinder provides a classification of whether the gene of interest is on the chromosome or a plasmid and provides the incompatibility group of the plasmid.

## Data Availability

The genome assemblies included in this study are available at the NCBI website (https://www.ncbi.nlm.nih.gov/bioproject, accessed on 1 November 2021) under BioProject PRJNA786273. The assembly accessions are listed in Table S1.

## Supplementary Materials

The following supporting information can be downloaded at https://www.mdpi.com/article/10.3390/antibiotics11060774/s1, Figure S1: (a) Biofilm growth over six days. The figure shows the absorbance at 570 nm of the crystal violet dye as a proxy for biofilm growth. Measurements were taken in duplicates on day 0 (red), day 3 (green), and day 6 (blue) for each isolate. (b) Representative image of bacterial biofilm growth. #1: mcr-positive E. coli growing in LB broth of day 0. #2: mcr-positive E. coli growing in LB broth of day 3. #3: Bacterial suspension of day 3 after being stained with crystal violet stain and measured at 570 nm using a spectrophotometer. #4: mcr-positive E. coli growing in LB broth of day 6. #5: Bacterial suspension of day 6 after being stained with crystal violet stain and measured at 570 nm using a spectrophotometer. Table S1: Genome accessions and sample sources.
