# Supplementary material for: Transmissibility and Persistence of the Plasmid-Borne Mobile Colistin Resistance Gene, mcr-1, Harbored in Poultry-Associated E. coli
Source: Antibiotics (Basel). 2022 Jun 7;11(6):774. doi: 10.3390/antibiotics11060774 (PMC9220209; doi:10.3390/antibiotics11060774)
Supplement: Supplementary file 1 [file antibiotics-11-00774-s001.zip › antibiotics-1724916-supplementary.pdf]

**Supplementary Materials**

**Table S1.** Genome accessions and sample sources

| Sample ID | BioSample Accession | Genome Accession | Sample Source |
|---|---|---|---|
| ad107 | SAMN23638422 | JAJOPA000000000 | Retail Chicken |
| ad108 | SAMN23638423 | JAJOPB000000000 | Retail Chicken |
| ad174 | SAMN23638424 | JAJOPC000000000 | Retail Chicken |
| ad28 | SAMN23638425 | JAJOPD000000000 | Retail Chicken |
| ad80 | SAMN23638426 | JAJOPE000000000 | Retail Chicken |
| ar181 | SAMN23638427 | JAJOPF000000000 | Retail Chicken |
| ar182 | SAMN23638428 | JAJOPG000000000 | Retail Chicken |
| br128 | SAMN23638429 | JAJOPH000000000 | Retail Chicken |
| br129 | SAMN23638430 | JAJOPI000000000 | Retail Chicken |
| cd62 | SAMN23638431 | JAJOPJ000000000 | Retail Chicken |
| cd63 | SAMN23638432 | JAJOPK000000000 | Retail Chicken |
| cr102 | SAMN23638433 | JAJOPL000000000 | Retail Chicken |
| cr103 | SAMN23638434 | JAJOPM000000000 | Retail Chicken |
| FC1 | SAMN23638435 | JAJOPN000000000 | Chicken Fecal |
| FC12 | SAMN23638436 | JAJOPO000000000 | Chicken Fecal |
| FC4 | SAMN23638437 | JAJOPP000000000 | Chicken Fecal |
| FC6 | SAMN23638438 | JAJOPQ000000000 | Chicken Fecal |
| FC7 | SAMN23638439 | JAJOPR000000000 | Chicken Fecal |

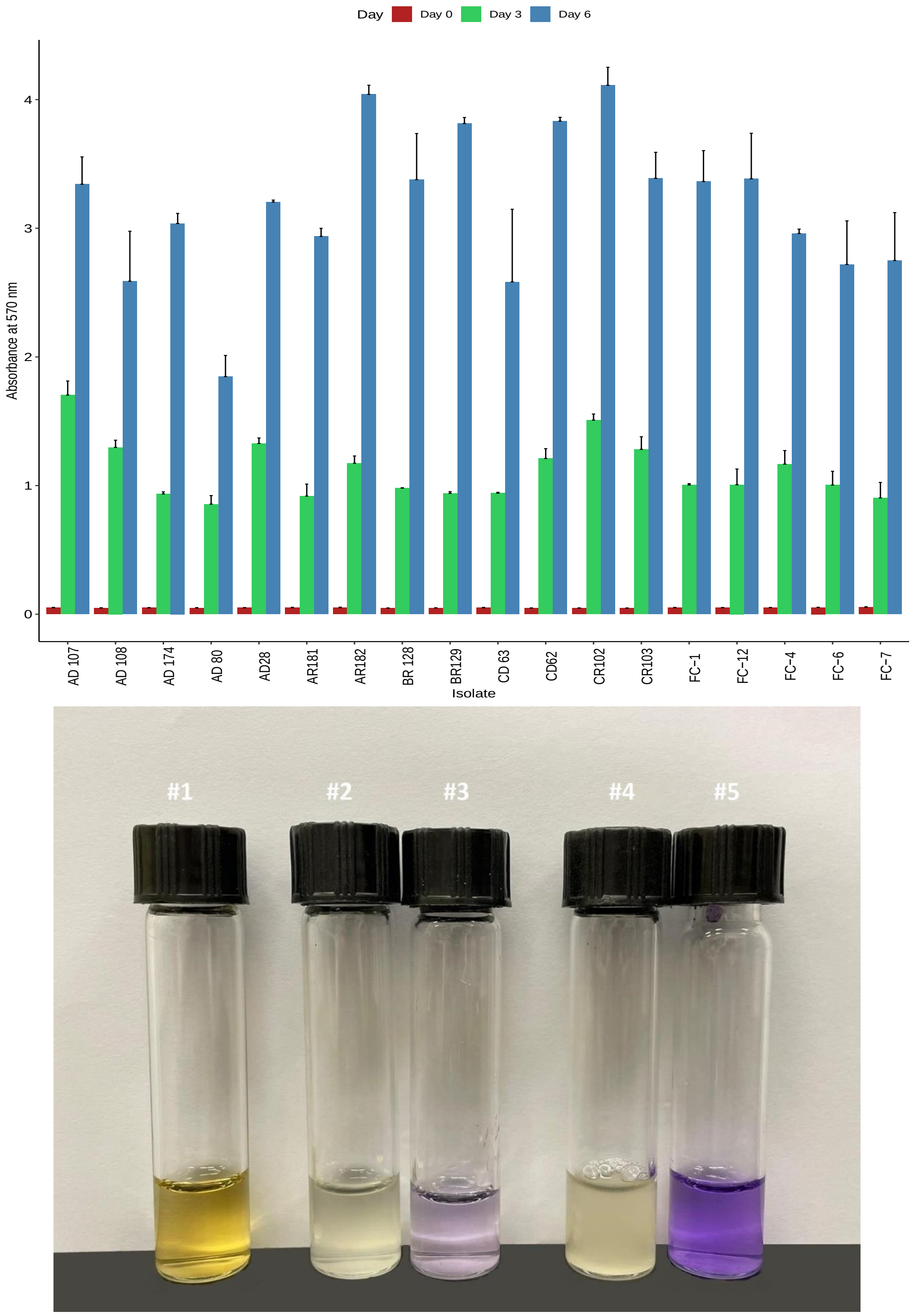


**(a)**

**(b)**

**Figure S1. (a) .** Biofilm growth over six days. The figure shows the absorbance at 570 nm of the Crystal violet dye as a proxy for biofilm growth. Measurements were taken in duplicates on day 0 (red), day 3 (green), and day 6 (blue) for each isolate. **(b).** Representative image of bacterial biofilm growth. #1: mcr- positive *E. coli* growing in LB broth of day 0. #2: mcr- positive *E. coli* growing in LB broth of day 3. #3: Bacterial suspension of day 3 after being stained with crystal violet stain and measured at 570 nm using a spectrophotometer. #4: mcr-positive *E. coli* growing in LB broth of day 6. #5: Bacterial suspension of day 6 after being stained with crystal violet stain and measured at 570 nm using a spectrophotometer.
